# 4396 Immunoglobulin administration and hypogammaglobulinemia during pediatric acute leukemia therapy

**DOI:** 10.1017/cts.2020.404

**Published:** 2020-07-29

**Authors:** Holly Edington, Shanmuganathan Chandrakasan, Tamara Miller, Nicholas DeGroote, Ann Mertens, Sharon Castellino

**Affiliations:** 1 Emory University; 2 Children’s Healthcare of Atlanta

## Abstract

OBJECTIVES/GOALS: Intravenous immunoglobulin (IVIG) is used for infection prevention in pediatric B-cell acute lymphoblastic leukemia (B-ALL), but evidence for this is lacking. We describe the prevalence of hypogammaglobulinemia in pediatric B-ALL, predictors of IVIG use and its efficacy for infection prevention. METHODS/STUDY POPULATION: We will conduct a retrospective review of children age 1-21 years with B-ALL treated at Aflac Cancer and Blood Disorders Center from 2010 to 2017. The cohort was identified through the cancer registry. Demographics, disease factors, laboratory values, medications and infection outcomes were linked between the electronic medical record and an institutional database. Outcomes of interest include emergency department (ED) visits, hospitalization days, and episodes of infection. Descriptive statistics will be performed. Outcomes will be compared between IVIG recipients and non-recipients. Univariate and multivariate logistic regression models will assess predictors of IVIG administration. RESULTS/ANTICIPATED RESULTS: We identified 443 patients with B-ALL during the study period who met inclusion criteria. Exclusion criteria included receipt of IVIG or hematopoietic stem cell transplant prior to diagnosis. The average age at diagnosis is 6.5 years (standard deviation 4.8 years); 52.6% are male; 61.6% are white; 61.0% are standard risk per National Cancer Institute criteria. Among eligible patients, 137 (31.1%) received IVIG. We hypothesize that IVIG initiation is associated with hypogammaglobulinemia and history of severe infection. We also anticipate that frequency of emergency department visits, hospitalization days, and episodes of infection will decrease after IVIG initiation. DISCUSSION/SIGNIFICANCE OF IMPACT: The immunological profile of children with B-ALL and factors influencing their susceptibility to infection are still incompletely understood. The benefits of IVIG are unknown. This study will provide evidence for IVIG prophylaxis recommendations in pediatric leukemia patients.

